# Surgical skills laboratories in plastic surgery residency training: A national survey of current practices and barriers

**DOI:** 10.1016/j.jpra.2026.07.004

**Published:** 2026-07-12

**Authors:** Israel Falade, Annie Chen-Carrington, Jacquelyn Knox, Daniel S. Rouhani, Chelsea Yim, Esther A. Kim

**Affiliations:** a Department of Surgery, Division of Plastic and Reconstructive Surgery, University of California San Francisco, San Francisco, CA, USA; b Division of Plastic and Reconstructive Surgery, Washington University, St Louis, MO, USA; c School of Medicine, University of California San Francisco, San Francisco, CA, USA; d School of Medicine, Loyola University Chicago Stritch School of Medicine, Maywood, IL, USA

**Keywords:** Plastic surgery residency, Surgical skills labs, Educational strategies, National survey, Competency based curriculum

## Abstract

**Background:**

Surgical skills labs (SSLs) provide plastic surgery residents hands-on experience in surgical techniques in a controlled learning environment. Despite growing evidence supporting simulation-based education, the adoption and structure of SSLs vary widely across plastic surgery residency programs. This study aimed to characterize the prevalence, organization, and funding of SSLs nationwide, identify barriers to implementation, and highlight strategies used by programs with established labs.

**Methods:**

A cross-sectional survey was distributed in February 2024 to program directors of all accredited U.S. plastic surgery residency programs. Programs were categorized by the presence or absence of dedicated SSL. Survey items addressed curriculum design, instructional content, participants, costs, administrative support, facilities, and perceived barriers.

**Results:**

Of 93 programs surveyed, 29 (31%) responded, with 19 reporting a dedicated SSL and 10 did not. Among programs with SSLs, 79% reported structured curricula developed through faculty and resident input. Common instructional topics included microvascular surgery (95%), fracture plating (79%), splinting (74%), non-surgical aesthetic procedures (74%), and cadaver-based dissections (56%). Thirteen programs used cadaver and non-cadaver sessions, while six relied solely on non-cadaver training. Funding sources included plastic surgery divisions (63%), industry (53%), and general surgery departments (32%). Programs without SSLs relied on periodic hands-on sessions and industry-sponsored workshops. Most frequently cited barriers to SSL implementation were funding constraints, limited faculty availability, and lack of dedicated facilities.

**Conclusion:**

Considerable variability exists in the availability, structure, and institutional support of surgical skills laboratories across plastic surgery residency programs. Survey responses from programs with established skills laboratories identified reproducible best practices including structured, modular curricula, designated faculty leadership, protected scheduling, strategic resource utilization, resident engagement, and embedded competency-based assessment that may guide future program development. Adoption of these practices, alongside collaborative and regional resource-sharing efforts, may help expand equitable access to high-quality simulation-based training nationwide.

## Introduction

Plastic surgery residency training requires progressive mastery of a broad spectrum of technical skills. Although operative experience remains the cornerstone of surgical education, increasing clinical demands and time constraints in the operating room may limit opportunities for deliberate practice and detailed formative feedback.^1^ Simulation-based education, particularly through surgical skills laboratories (SSLs), has emerged as a critical adjunct to operative training, allowing residents to develop and refine technical skills in a low-stakes environment.^2^, ^3^, ^4^

SSLs are designed to approximate operative conditions using cadaveric, animal, synthetic, and virtual models.^5^ Through repeated practice and structured feedback, residents can strengthen anatomical understanding, improve dexterity and efficiency, and gain confidence prior to performing procedures in the operating room.^6^, ^7^, ^8^ Simulation-based training has been shown to improve technical performance across multiple surgical disciplines and has been incorporated widely into general surgery training through standardized curricula such as Fundamentals of Laparoscopic Surgery (FLS) and Fundamentals of Endoscopic Surgery (FES).^9^

Within plastic surgery, however, the implementation of SSLs remains heterogenous. Prior studies suggest that resource limitations, competing educational demands, and lack of standardized curricula impede widespread adoption.^10^, ^11^, ^12^, ^13^, ^14^, ^15^, ^16^, ^17^, ^18^ A comprehensive understanding of the current landscape of plastic surgery SSLs is needed to inform efforts toward broader implementation and standardization. Accordingly, this national survey sought to describe the prevalence and characteristics of SSLs in U.S. plastic surgery residency programs, identify perceived barriers to implementation, and highlight strategies employed by programs with established labs.

## Methods

### Survey development and content

This study was approved by our institutional review board (#24–40,778). A cross-sectional electronic survey was designed using Qualtrics platform. Survey items were created through iterative discussion among the study authors and pilot-tested by one program director and two surgical residents to optimize clarity and content validity. The complete survey instrument is provided in Supplementary Material 1.

The survey distinguished between programs with and without dedicated skills labs and comprised a mix of multiple-choice and open-ended questions. For those programs with a dedicated SSL, items addressed curriculum design, instructional topics, learner and faculty participation, costs, facilities, access, and administrative oversight. For programs without SSL, items explored alternative educational strategies and perceived barriers to establishing a formal lab.

### Participants and survey administration

All accredited U. S. integrated plastic surgery residency programs were identified. Program directors were contacted via publicly available institutional email addresses in February 2024 and invited to complete the survey or forward it to a knowledgeable faculty designee. Reminder emails were sent at 1, 2, and 3 week intervals. Responses were collected anonymously, and the survey closed in April 2024.

### Data and statistical analysis

Survey data were exported to a secure database and analyzed using Stata version 17.0 (College Station, TX, USA). Only IRB-approved study personnel were allowed access to the data. Descriptive statistics were used to summarize program characteristics and survey responses.

## Results

### Characteristics of programs with a dedicated skills lab

Curriculum & Development: Of the 93 programs surveyed, 29 (31%) responded, with 19 reporting a dedicated SSL and 10 lacking a dedicated SSL. Among the programs with SSL, 79% described a formal curriculum developed through faculty and resident collaboration. Curriculum effectiveness was assessed using satisfaction surveys (37%) and competency assessment tools (32%).

Instructional Content & Scheduling: Microvascular surgery was the most frequently included topic (95%), followed by fracture plating (79%), splinting lab (74%), nonsurgical aesthetic procedure (74%), and cadaveric sessions (56%) (Table 1). Thirteen programs utilized both cadaveric and non-cadaveric sessions, while six relied exclusively on non-cadaveric models. Lab sessions occurred weekly (11%), monthly (21%), quarterly (32%), biannually (5%), annually (21%), or as-needed (11%) (Table 2). Sessions ranged from 1 to 6 h with an average of 2.8 h in duration (Table 2).

**Table 1:** Skills lab modules in plastic surgery residency programs.

Module	Programs (n = 19)
Microvascular surgery	18 (95%)
Fracture plating lab (Craniofacial or Hand-related)	15 (79%)
Non-surgical aesthetic procedures (e.g., Botox and Fillers)	14 (74%)
Splinting lab	14 (74%)
Upper extremity/hand reconstruction	13 (69%)
Lower extremity reconstruction	11 (58%)
Craniofacial surgery	11 (58%)
Trunk and abdominal wall reconstruction	10 (53%)
Advanced wound closure (rotation, advancement, other local flaps, etc.)	9 (47%)
Instrument handling, knot tying, suturing	9 (47%)
Gender affirming surgery	4 (21%)

**Table 2:** Skills lab frequency.

Frequency	Programs (n = 19)
Weekly	2 (11%)
Monthly	4 (21%)
Quarterly	6 (32%)
Biannually	1 (5%)
Annually	4 (21%)
By appointment	2 (11%)

**Fig. 1:**
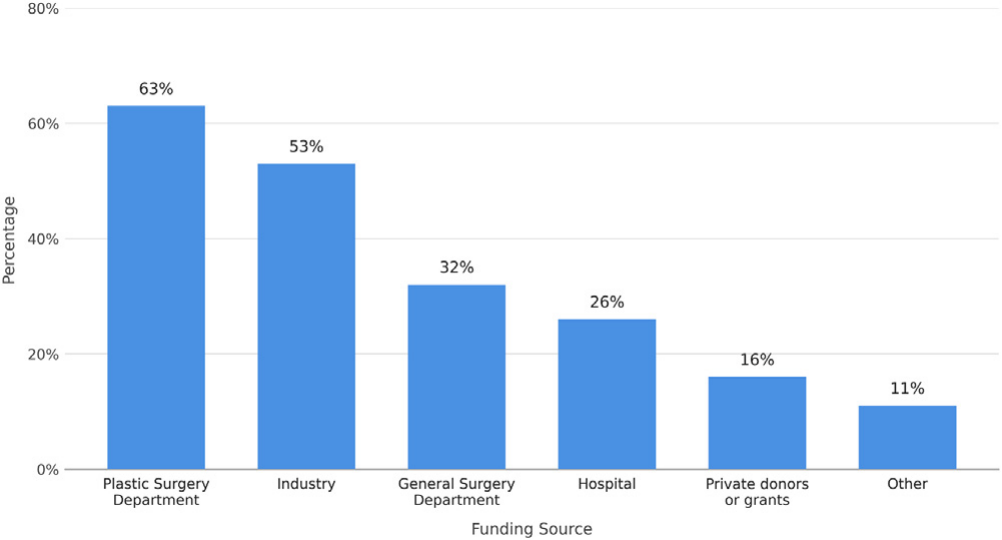
Funding sources for plastic surgery skills labs.

Funding and Resources: Estimated start-up costs ranged from $5000 to several million dollars with annual operating budgets between $5000 to $300,000. Primary funding sources included plastic surgery divisions (63%), industry sponsorship (53%), and general surgery departments (32%) (Fig. 1). Most programs (68%) shared simulation space with other departments and medical school, while 32% reported exclusive access to surgical residents. Only one program reported dedicated full-time staff support for SSL operations. Access to the lab varied, 32% of programs offering 24-hour access to the skills labs without prior reservation, 26% requiring reservations for extended use, and 42% limiting access to designated hours. The most commonly available equipment was surgical instruments (95%), and microscopes (79%), whereas only one skills lab had animal models (5%).

Leadership and Perception: SSLs were overseen most commonly by program directors, associate program directors, or designated faculty. Ninety percent of respondents viewed SSLs as an essential component of residency education, and 42% supported the development of a national competency-based skills lab curriculum. Furthermore, these program directors recommended that programs lacking dedicated skills labs should consider industry sponsors as a key resource for securing funding for skills lab start-up costs.

### Characteristics of programs without a dedicated skills lab

Barriers & Alternatives: Among the 10 programs that reported no dedicated SSL, six had considered establishing one. They identified securing grant funding and access to specialized faculty and trainers as vital prerequisites for setting up a skills lab. Additional obstacles cited to formalizing a lab were funding (70%), faculty availability (60%), the lack of facilities (40%) and limited faculty interest (40%). In lieu of a dedicated lab, programs relied on periodic hands-on sessions (80%), industry-sponsored workshops (50%), and external courses (70%).

Perceptions: When asked about their perceptions of skills laboratories, seven of the programs without a dedicated skills lab felt that a structured skills curriculum would benefit their plastic surgery residency program, and five agreed that a national competency-based skills lab curriculum would also be advantageous.

## Discussion

This national survey provides a snapshot of how surgical skills labs (SSLs) are incorporated into U.S. plastic surgery residency training and highlights both progress and persistent gaps in simulation-based education. Although a majority of responding programs reported access to a dedicated SSL, substantial variability exists in curricular content, frequency, funding mechanisms, and institutional support. These differences likely translate into unequal educational opportunities for residents and underscore the absence of a shared national framework guiding skills lab development in plastic surgery.

### Variability and educational equity

One of the most striking findings of this study is the heterogeneity in SSL availability and structure. While some programs reported robust, longitudinal curricula with regular sessions, dedicated faculty leadership, and multimodal assessment, others relied on sporadic workshops or external courses to supplement operative experience. Such variability raises concerns regarding educational equity across residency programs, particularly as simulation-based training increasingly serves as a prerequisite for operative readiness. Residents training in programs without reliable access to SSLs may have fewer opportunities for deliberate practice, structured feedback, and remediation outside of the operating room.

This variability mirrors earlier findings in plastic surgery education but remains notable given growing national emphasis on competency-based training and patient safety.^4^^,^^19^ As operative autonomy becomes increasingly scrutinized, SSLs offer a controlled environment to ensure baseline technical competence prior to independent clinical performance. Without greater standardization, however, the educational benefits of simulation risk being unevenly distributed.

### Barriers to implementation and sustainability

Programs without dedicated SSLs most frequently cited funding limitations, faculty availability, and lack of appropriate facilities as barriers to implementation. These challenges are consistent with reports from other surgical specialties and highlight the resource intensive nature of simulation-based education.^2^^,^^3^^,^^9^ Start-up costs for infrastructure and equipment, particularly microscopes and cadaveric resources can be prohibitive, especially for smaller programs or those without institutional simulation centers.

Faculty time represents another critical barrier. The absence of protected teaching time or financial incentives for faculty participation was common among surveyed programs, even those with established SSLs. This finding underscores the need for departmental and institutional recognition of skills lab teaching as a core educational responsibility. Without dedicated support, SSLs may rely on a small number of motivated faculty, threatening long-term sustainability.

### Curricular focus and opportunities for standardization

Despite broad variability, several areas of curricular convergence emerged. Microsurgery was included in nearly all SSL curricula, reflecting its central role in plastic surgery training and its suitability for simulation-based instruction. This near-universal emphasis suggests that microsurgery represents a logical entry point for national curricular standardization. Analogous to the Fundamentals of Laparoscopic Surgery (FLS) and Fundamentals of Endoscopic Surgery (FES) curricula in general surgery, a standardized Fundamentals of Microsurgery (FMS) curriculum could provide a scalable, competency-based framework adaptable to diverse institutional resources.^20^^,^^21^ These curricula include both didactic and web-based material, as well as a simple, affordable physical simulator with specific tasks.

Existing models demonstrate that effective microsurgical training can be achieved using low-cost or portable simulation tools and smartphone technology, reducing reliance on expensive operating microscopes and cadaveric specimens.^22^ Other attempts to establish microsurgery curricula include a proposed six-year competency-based FMS curriculum,^23^ a 12-week FMS program utilizing chicken femoral arteries to improve needle dexterity, the economy of movement, operative flow, and operative judgment,^24^ and other programs in orthopedic and transplant surgery that demonstrated significant improvements for microsurgery training in residency education.^25^^,^^26^ Broader adoption of such approaches may help lower barriers for programs currently unable to support comprehensive SSLs.

Importantly, respondents expressed interest in national guidance, with both programs with and without SSLs endorsing the value of a competency-based skills lab curriculum. This alignment suggests readiness within the specialty for collaborative curriculum development under the auspices of national organizations.

### Best practices and programmatic implications

Survey responses from programs with established skills laboratories revealed several best practices that may inform future plastic surgery residency program development (Table 3). Across programs with established SSLs, several recurring structural and pedagogical elements emerged that appear critical to effectiveness, scalability, and longevity. These best practices provide a practical roadmap for programs seeking to develop new skills labs or strengthen existing ones, regardless of resource constraints.

**Table 3:** Best practices for plastic surgery skills labs.

Category	Best Practice
Curriculum Development	Utilize a structured, modular curriculum which can cover core skills which are tailored to address specific training needs based on gaps in clinical exposure.
Faculty Engagement	Ensure faculty buy in with departmental support and protected teaching time. Designate one faculty member as the skills lab leader to oversee curriculum, organize sessions, and manage resources.
Scheduling and Session Structure	Establish consistent, scheduled lab time, ideally as half-day sessions each month, to allow for regular practice without significant disruption to clinical duties. Organize sessions by resident experience level for targeted learning.
Funding and Resource Allocation	Pursue diverse funding sources, including department budgets, industry sponsorships, and school of medicine resources. Optimize costs through shared use of cadaver labs, simulation spaces, and surgical instruments across departments.
Resident Engagement	Encourage resident participation through the use of preparatory assignments before each skills lab. Utilize independent practice modules to foster proactive skill-building.
Evaluation and Assessment	Implement competency-based assessment tools to track resident progress. Use clear performance metrics and milestones to provide structured, actionable feedback that guides skill development.

Categories represent a descriptive synthesis of recurring practices reported by responding programs, not a formal qualitative analysis.

**1. Intentional Curriculum Design and Modularity**

High-functioning SSLs consistently employed structured, modular curricula rather than ad hoc or opportunistic sessions. Modular design allows programs to target discrete competencies such as microsurgical anastomosis, fracture fixation, or local flap design while accommodating variability in resident experience and institutional case mix. Programs reported greater perceived value when curricula were explicitly mapped to anticipated clinical exposure gaps, ensuring that simulation complemented, rather than duplicated, operative experience. Importantly, modular curricula also facilitate incremental implementation, allowing programs to begin with a limited scope and expand as resources permit.

**2. Designated Faculty Leadership and Departmental Buy-In**

A defining feature of successful SSLs was the presence of a designated faculty champion responsible for curricular oversight, scheduling, coordination of resources, and longitudinal refinement of the lab. Programs without clear leadership often described fragmented or inconsistent skills lab offerings. Departmental buy-in including recognition of SSL teaching as a core educational responsibility and allocation of protected faculty time was strongly associated with sustainability. Without institutional support, skills labs were more vulnerable to cancellation, inconsistent participation, and faculty burnout.

**3. Protected Time and Predictable Scheduling**

Programs reporting the highest satisfaction with their SSLs emphasized consistent, protected scheduling most commonly monthly or quarterly half-day sessions. Predictable scheduling minimized conflicts with clinical duties and reinforced the perception of skills labs as an integral component of residency training rather than an optional add-on. Several programs also described stratifying sessions by postgraduate year, allowing junior residents to focus on foundational skills while senior residents engaged in advanced or procedure-specific simulations. This tiered approach maximized educational efficiency and relevance.

**4. Strategic Use of Resources and Cost Optimization**

Given the financial barriers identified, successful programs demonstrated flexibility and creativity in resource utilization. Common strategies included shared use of cadaver labs and simulation spaces with other departments, reliance on reusable synthetic models, and selective incorporation of cadaveric sessions for high-yield or anatomically complex topics. Industry partnerships were frequently cited as instrumental in supporting equipment acquisition and consumable supplies, particularly during program start-up. Importantly, programs emphasized aligning resource investment with educational priorities rather than attempting to build comprehensive facilities at the outset.

**5. Active Resident Engagement and Ownership**

Resident involvement emerged as a critical driver of SSL relevance and success. Programs that engaged residents in curriculum development, session planning, or peer teaching reported higher participation and perceived educational value. Preparatory assignments, pre-lab videos, and independent practice modules further enhanced engagement by allowing residents to arrive prepared for deliberate practice rather than passive participation. This bidirectional model fostered a culture of continuous improvement and accountability within the skills lab.

**6. Embedded Evaluation and Feedback Mechanisms**

Finally, effective SSLs integrated structured assessment tools to support formative feedback and longitudinal tracking of skill acquisition, consistent with prior work emphasizing objective assessment in plastic surgery training.^27^ Programs using competency-based metrics such as global rating scales or task-specific checklists reported greater clarity in resident progression and remediation needs. Assessment data were most impactful when used to guide individualized feedback and inform future curriculum adjustments rather than as isolated performance measures. Embedding evaluation within the skills lab reinforced its role as both a teaching and assessment environment.

Collectively, these best practices underscore that successful skills labs are not defined solely by physical infrastructure or financial investment. Rather, they depend on intentional educational design, leadership commitment, and integration into the broader residency curriculum. Even programs without dedicated facilities can adopt many of these principles to enhance simulation-based training incrementally and sustainably.

### Integration with competency-based assessment and EPAs

Beyond skill acquisition, SSLs offer a unique platform for competency-based assessment. The structured nature of skills labs, defined tasks, standardized conditions, and direct observation, aligns well with entrustable professional activities (EPAs). Integrating EPA-aligned milestones into SSL curricula may allow programs to document resident progression more objectively, link simulation performance to operative readiness, and support Clinical Competency Committee decision-making. Evidence from other health professions suggests that EPA-based assessment within simulation environments improves feedback quality and supports longitudinal development.^28^ In plastic surgery, such integration could help bridge the gap between simulated performance and real-world operative entrustment, reinforcing the educational value of SSLs beyond technical rehearsal alone.

### Limitations

This study has several limitations. The 31% response rate, while modest, is consistent with prior national surveys of plastic surgery program directors, including a 31.6% response rate reported by Powell et al.^29^ and a 50% response rate reported by Al-Bustani and Halvorson in an earlier U.S. survey of microsurgical simulation training.^30^ Likely contributors to the modest response rate include survey fatigue, competing administrative demands among program directors, and the length of the instrument. Because survey responses were collected anonymously, we were unable to compare institutional or demographic characteristics between responding and non-responding programs, which limits our ability to fully characterize the risk of non-responder bias. It is possible that programs with more developed or well-resourced skills laboratories were more likely to participate, which may overstate how established SSLs are nationwide. Data were self-reported and may be subject to recall bias or incomplete knowledge of program resources. Future mixed-methods studies incorporating resident perspectives and qualitative interviews may provide additional insight.

## Conclusion

This national survey demonstrates that surgical skills laboratories are widely regarded as a critical component of plastic surgery residency education, yet access, structure, and institutional support vary substantially across programs. These disparities reflect differences in funding, faculty availability, facilities, and curricular infrastructure, and may contribute to unequal educational experiences among trainees nationwide.

Importantly, survey responses from programs with established skills laboratories identified a set of best practices that transcend institutional resources and may guide future program development. These include intentional, modular curriculum design; designated faculty leadership with departmental support; protected and predictable scheduling; strategic use of shared and cost-efficient resources; active resident engagement in curriculum development; and embedded competency-based assessment. Collectively, these practices emphasize that successful skills laboratories are defined not solely by physical infrastructure, but by thoughtful educational design and integration within the residency curriculum.

In the absence of a nationally standardized competency-based skills curriculum in plastic surgery, these best practices offer a pragmatic framework for programs seeking to establish or strengthen simulation-based training. Similar to the role of the Fundamentals of Laparoscopic Surgery curriculum in general surgery, development of standardized, modular curricula particularly in microsurgery represents a feasible and impactful first step toward broader national alignment.

By highlighting both existing gaps and reproducible strategies for success, this study provides actionable guidance for residency programs, educators, and professional societies aiming to advance equitable, competency-based training in plastic surgery.

## Funding

None.

## Ethical approval

Approved by the University of California, San Francisco IRB (#24-40,778).

## Financial disclosure statement

The authors did not receive support from any organization for the submitted work.

## Declaration of competing interest

None declared.

## Appendix Supplementary materials

Image, application 1
