# Supplementary material for: Surgical skills laboratories in plastic surgery residency training: A national survey of current practices and barriers
Source: JPRAS Open. 2026 Jul 12;51:511–9. doi: 10.1016/j.jpra.2026.07.004 (PMC13448203; doi:10.1016/j.jpra.2026.07.004)
Supplement: Supplementary file 1 [file mmc1.docx]

**Supplemental Material 1**

**Survey Instrument**

*Surgical Skills Laboratories in Plastic Surgery Residency Training: A National Survey of Current Practices and Barriers*

The following survey was administered electronically through Qualtrics in February 2024 to program directors of all accredited U.S. integrated plastic surgery residency programs. The survey contained branching logic: respondents indicating their program had a dedicated surgical skills laboratory (SSL) were directed to Section A; those indicating no dedicated SSL were directed to Section B.

**Introduction and Screening**

**1.** Would you be willing to complete a survey to help us establish a baseline understanding of current skills laboratories? (~10 minutes)

○ Yes

○ No

**2.** Does your program hold “formal” skills laboratory sessions with a structured curriculum for plastic surgery residents?

*For example: a program that holds regular skills labs at a designated space(s) with frequently repeating topics from year to year would qualify. A program that holds infrequent, or “one off” skills sessions that frequently do not repeat from year to year would not qualify.*

○ Yes (comments may be entered below for clarification if needed)

○ No

*[If Yes, optional comments]*

*Branching logic: If Yes, proceed to Section A. If No, skip to Section B.*

**Section A. Programs with a Dedicated Skills Laboratory**

***Schedule and Curriculum***

**A1.** Approximately how many skills lab sessions are held per academic year?

*[Numeric response]*

**A2.** Approximately how often are skills labs held?

○ Weekly

○ Monthly

○ Quarterly

○ Other (describe below)

*[If Other, please describe]*

**A3.** On average, how long are your skills lab sessions (in hours)?

*[Numeric response]*

**A4.** Do you have a set skills lab curriculum for your program?

○ Yes

○ No

**A5.** Which of the following methods were used to inform the design of your skills lab curriculum? (select all that apply)

☐ Informal discussion with faculty and/or residents

☐ Formal interviews with faculty and/or residents

☐ Focus group discussions with faculty and/or residents

☐ Questionnaires sent out to faculty and/or residents

☐ Test results (In service exams, internal exams, skills assessments, etc.)

☐ Published guidelines or literature (please describe)

☐ Other (please describe)

*[If Published guidelines or Other, please describe]*

**A6.** What modules are taught at your skills laboratory? (select all that apply)

☐ Instrument handling, knot tying, suturing

☐ Advanced wound closure (rotation, advancement, other local flaps, etc.)

☐ Splinting lab

☐ Fracture plating lab (Craniofacial or Hand related)

☐ Upper extremity/hand reconstruction

☐ Lower extremity reconstruction

☐ Trunk and abdominal wall reconstruction

☐ Microvascular surgery

☐ Craniofacial surgery

☐ Aesthetic surgery/procedures (e.g., Botox and fillers)

☐ Gender affirming surgery

☐ Other (describe below)

*[If Other, please describe]*

**A7.** What assessment measures are used in the skills lab? (select all that apply)

☐ Competency assessments

☐ Satisfaction or confidence surveys

☐ Anonymous feedback

☐ None

☐ Other (describe below)

*[If Other, please describe]*

**A8.** Do you think that all plastic surgery residency programs should include skills labs as part of the educational curriculum? Why or why not?

*[Open-ended response]*

**A9.** In your opinion, would the plastic surgery residency programs benefit from a national competency-based skills lab curriculum? This curriculum would aim to ensure consistent training standards across all programs, focusing on core competencies and skills essential for plastic surgery residents.

○ Yes (feel free to add comments below)

○ No

○ Unsure

*[If Yes, optional comments]*

***Funding and Resources***

**A10.** What were the approximate start-up costs to establish your skills lab (in dollars)? Please give your best estimate.

○ Dollar amount: ____________

○ Unsure

**A11.** What were the start-up costs used for? (e.g., building a simulation center, hiring skills lab administrators, purchasing skills lab materials, cadavers, room rentals, etc.)

*We aim to share this information with other programs hoping to start a skills laboratory, so please be as detailed as possible in providing a cost breakdown for this question.*

*[Open-ended response]*

**A12.** From what sources did you receive start-up funding for establishing the skills laboratory? (select all that apply)

☐ Industry

☐ Surgery Department

☐ Plastic Surgery Department/Division

☐ Hospital

☐ Private donors or grants

☐ Other (describe below)

*[If Other, please describe]*

**A13.** What is the approximate annual budget for your skills lab, excluding the start-up costs (in dollars)? Please give your best estimate.

○ Dollar amount: ____________

○ Unsure

**A14.** From what sources do you receive funding for your annual budget? (select all that apply)

☐ Industry

☐ General Surgery Department

☐ Plastic Surgery Department

☐ Hospital

☐ Private donors or grants

☐ Other (describe below)

*[If Other, please describe]*

**A15.** Do you have any recommendations or advice about obtaining funding for your skills laboratory to share with those hoping to start a new skills laboratory at their program? (e.g., specific grant mechanisms, industry partnerships, etc.)

*[Open-ended response]*

***Faculty Leadership and Staffing***

**A16.** Which faculty member is in charge of developing the content and overall curriculum for the skills lab sessions? (While additional faculty may provide support, we are interested in identifying the key faculty member leading this responsibility.)

○ Program Director

○ Associate Program Director

○ Other faculty surgeon

○ Other (describe below)

*[If Other, please describe]*

**A17.** Is there dedicated time (% FTE) allocated for this key faculty member to run the skills lab?

○ Yes

○ No

○ Unsure

**A18.** How much time as a percentage FTE? (You may also indicate unsure or prefer not to answer.)

*[Numeric or open-ended response]*

**A19.** Who facilitates the skills labs? (select all that apply)

☐ Faculty

☐ Non-faculty surgeons (invited guests)

☐ Industry educators

☐ Administrative staff

☐ Other (describe below)

*[If Other, please describe]*

**A20.** On average, how many facilitators, capable of providing teaching or hands-on assistance with lab content, are present at each skills lab session?

*[Numeric response]*

**A21.** What types of incentives are given to faculty to teach skills labs? (select all that apply)

☐ No incentives

☐ Other (describe below)

*[If Other, please describe]*

**A22.** Are there administrative support personnel who regularly assist the key faculty member with the skills lab (i.e., lab technician or lab coordinator)?

○ Yes (please describe)

○ No

*[If Yes, please describe]*

**A23.** Does your program or hospital offer skills lab facilitators formal instruction sessions on teaching?

○ Yes, it is required in order to teach in the skills lab

○ It is offered, but not required

○ No, there is no formal instruction on teaching

○ Unsure

○ Other (describe below)

*[If Other, please describe]*

**A24.** You indicated that formal instruction sessions on teaching are offered or required. What are these sessions?

*[Open-ended response]*

***Facilities, Access, and Equipment***

**A25.** Is the skills lab space exclusively dedicated to your plastic surgery program and other surgical departments (e.g., general surgery, neurosurgery), or is it shared with other non-surgical departments (e.g., emergency medicine, medical school students, nursing students)?

○ Dedicated to surgery residents

○ Shared with other departments/students

**A26.** Where is your skills lab held? Please be as specific as possible. (Examples: in a dedicated simulation center, in the medical school's anatomy lab, on the medical campus, off the medical campus, etc.)

*[Open-ended response]*

**A27.** What type of access do you have to the skills laboratory?

○ 24-hour access (no reservation necessary)

○ 24-hour access (with reservation or appointment)

○ During designated hours only

**A28.** What skills laboratory equipment is provided for use at baseline (always available)? (check all that apply)

☐ Surgical instruments

☐ Microscope

☐ Sawbone models

☐ Power tools and plating sets (i.e., for Hand and Craniofacial surgery)

☐ Cadavers

☐ Animal models

☐ Other (describe below)

*[If Other, please describe]*

**A29.** What skills laboratory equipment can be obtained through special request or at an additional cost (not always available)? (check all that apply)

☐ Surgical instruments

☐ Operating microscope

☐ Sawbone models

☐ Power tools and plating sets (i.e., for Hand and Craniofacial surgery)

☐ Cadavers

☐ Animal models

☐ Other (describe below)

*[If Other, please describe]*

***Program Demographics***

**A30.** What kind of program are you in?

○ Academic

○ Community

○ Other (describe below)

*[If Other, please describe]*

**A31.** How many residents (integrated and independent) graduate from your program each year?

*[Numeric response]*

**A32.** Do you have any final comments?

*[Open-ended response]*

**A33.** Please feel free to include any attachments with additional information you'd feel comfortable sharing (budget documents, curriculum, etc.). Please remove any identifying information you'd like not to share.

*[File upload (optional)]*

**Section B. Programs without a Dedicated Skills Laboratory**

***Program Demographics***

**B1.** What kind of program are you in?

○ Academic

○ Community

○ Other (describe below)

*[If Other, please describe]*

**B2.** How many residents (integrated and independent) graduate from your program each year?

*[Numeric response]*

***Barriers and Alternatives***

**B3.** Has your program ever considered establishing a skills laboratory in the past?

○ Yes

○ No

○ Unsure

**B4.** What is the barrier to having a skills laboratory? (select all that apply)

☐ Funding

☐ Faculty availability

☐ Faculty interest

☐ Resident interest

☐ Adequate space or facilities

☐ Other (describe below)

*[If Other, please describe]*

**B5.** In the absence of a formal skills laboratory, what approaches does your program utilize to supplement surgical skills training? These approaches may address any gaps in hands-on experience or exposure to certain procedures that residents may not typically encounter in the operating room or during regular clinical practice. (select all that apply)

☐ Periodic hands-on training sessions or workshops

☐ Industry-sponsored workshops

☐ Sponsored attendance to external surgical workshops or courses (e.g., Duke flap course, Penn flap course)

☐ Elective rotations or away rotations with different surgical specialties

☐ Online tutorials and video demonstrations

☐ Simulation tools and models

☐ Other (describe below)

*[If Other, please describe]*

**B6.** You indicated external surgical workshops are sponsored. Which external workshops or courses are sponsored for residents to attend?

*[Open-ended response]*

***Periodic Hands-On Training Sessions (if applicable)***

**B7.** You indicated periodic hands-on training sessions or workshops are held. Approximately how many of these sessions are held per year?

*[Numeric response]*

**B8.** Who facilitates the periodic hands-on training sessions or workshops? (select all that apply)

☐ Faculty

☐ Non-faculty surgeons

☐ Industry educators

☐ Other (describe below)

*[If Other, please describe]*

**B9.** What is taught during the periodic hands-on training sessions or workshops? (select all that apply)

☐ Instrument handling, knot tying, suturing

☐ Advanced wound closure (rotation, advancement, other local flaps, etc.)

☐ Splinting lab

☐ Fracture plating lab (Craniofacial or Hand related)

☐ Upper extremity/hand reconstruction

☐ Lower extremity reconstruction

☐ Trunk and abdominal wall reconstruction

☐ Microvascular surgery

☐ Craniofacial surgery

☐ Aesthetic surgery/procedures (e.g., Botox and fillers)

☐ Gender affirming surgery

☐ Other (describe below)

*[If Other, please describe]*

**B10.** How are these periodic hands-on training sessions or workshops funded? (select all that apply)

☐ Industry

☐ Surgery Department

☐ Plastic Surgery Department/Division

☐ Hospital

☐ Private donors or grants

☐ Other (describe below)

*[If Other, please describe]*

***Skills Assessment and Perceptions***

**B11.** How do you assess residents' surgical skills and proficiency outside of a structured skills curriculum? (select all that apply)

☐ Real-time constructive feedback from faculty

☐ Formal faculty evaluation of resident

☐ Resident self-assessment surveys

☐ Formal competency assessment (please specify what assessment tool is utilized)

☐ Other (describe below)

*[If Formal competency assessment, please specify the tool utilized]*

*[If Other, please describe]*

**B12.** Do you believe that a structured skills curriculum would benefit your plastic surgery residency program?

○ Yes

○ No

○ Unsure

**B13.** In your opinion, would plastic surgery residency programs benefit from a national competency-based skills lab curriculum? This curriculum would aim to ensure consistent training standards across all programs, focusing on core competencies and skills essential for plastic surgery residents.

○ Yes (feel free to add comments below)

○ No

○ Unsure

*[If Yes, optional comments]*

**B14.** What resources or support would be most helpful to establish a structured skills lab in the future? (select all that apply)

☐ Financial support or grants

☐ Faculty or trainers specialized in surgical skills

☐ Skills lab technicians or additional staff

☐ Space and infrastructure support

☐ Access to surgical tools and equipment

☐ An established national curriculum

☐ Other (describe below)

*[If Other, please describe]*

**B15.** Do you have any final comments?

*[Open-ended response]*

*— End of Survey —*
